# Role of Cytokines in Thymic Regulatory T Cell Generation: Overview and Updates

**DOI:** 10.3389/fimmu.2022.883560

**Published:** 2022-03-31

**Authors:** Mei Tang, Fuya Jia, Fang Nan, Fengqiong Zuo, Zhu Yuan, Dunfang Zhang

**Affiliations:** ^1^ Department of Biotherapy, State Key Laboratory of Biotherapy and Cancer Center, West China Hospital, Sichuan University, Chengdu, China; ^2^ West China School of Pharmacy, Sichuan University, Chengdu, China; ^3^ Department of Immunology, West China School of Basic Medical Sciences and Forensic Medicine, Sichuan University, Chengdu, China; ^4^ State Key Laboratory of Biotherapy and Cancer Center, West China Hospital, Sichuan University, Chengdu, China

**Keywords:** tTreg cells, IL-2, IL-15, TGF-β, γc family cytokines, TNF superfamily, TNFRSF

## Abstract

CD4^+^CD25^+^Foxp3^+^ Regulatory (Treg) T cells are mainly generated within the thymus. However, the mechanism of thymic Treg cell (tTreg cell) generation remains to be fully revealed. Although the functions of TCR/CD28 co-stimulation have been widely accepted, the functions of cytokines in the generation of tTreg cells remain highly controversial. In this review, we summarize the existing studies on cytokine regulation of tTreg cell generation. By integrating the key findings of cytokines in tTreg cell generation, we have concluded that four members of γc family cytokines (IL-2, IL-4, IL-7 and IL-15), transforming growth factor β (TGF-β), and three members of TNF superfamily cytokines (GITRL, OX40L and TNF-α) play vitally important roles in regulating tTreg cell generation. We also point out all disputed points and highlight critical scientific questions that need to be addressed in the future.

## Introduction

CD4^+^ Regulatory T (Treg) cells that express IL-2 receptor α-chain (CD25) and the transcription factor forkhead box P3 (Foxp3) are the major cell population that maintains immune tolerance (1–6). Since these cells were identified in 1995 (2), Treg cells have been demonstrated to play extremely important roles in maintaining tolerance to auto-antigens (7, 8) and commensal microbiota (9, 10), controlling maternal-fetal immune interactions (11, 12), and suppressing overactive immune responses during infection (13, 14). On the other hand, Treg cell-mediated immune suppression can also promote tumor immune escape (15, 16). Therefore, targeting Treg cells could be a promising strategy to treat autoimmune disorders, maternal-fetal conflict, infections, and malignant tumors.

A majority of Treg cells are generated in the thymus (thymic Treg cells, tTreg cells), however some Treg cells can also be generated in periphery (pTreg cells) (17). Although it has been well documented that tTreg cells are generated during CD4^+^ thymocyte development, the clear mechanisms of tTreg cell development is still not completely understood. Since T-cell receptor (TCR) stimulation from self-antigens and CD28 co-stimulation during thymocyte development are indispensable for tTreg cell generation (18–20), the mainstream view once believed that high-affinity TCR signal is the main driving force for inducing Treg cell differentiation (21–23). However, later studies demonstrated that tTreg cells could be generated from developing CD4^+^ thymocytes expressing TCRs with a broad range of self-reactivity (24, 25), showing that the self-reactivity of the TCR signal is not the deciding factor for tTreg cell generation.

In contrast, a two-step model of tTreg cell generation is gaining acceptance (26–29). The first step is driven by self-antigen induced TCR stimulation and CD28 co-stimulation, which leads to differentiation of CD4^+^ CD8^-^ Foxp3^-^ CD25^+^ tTreg cell precursors (CD25^+^ Foxp3^-^ tTreg precursors) and CD4^+^ CD8^-^ Foxp3^+^ CD25^-^ Treg cell precursors (Foxp3^+^ CD25^-^ tTreg precursors) from developing CD4^+^ CD8^-^ thymocytes. The second step relies on IL-2, which leads to the generation of CD25^+^Foxp3^+^ mature tTreg cells from CD25^+^ Foxp3^-^ tTreg precursors and Foxp3^+^ CD25^-^ tTreg precursors. This model proposes that both precursor populations are induced by TCR/CD28 co-stimulation, and both precursor populations rely on IL-2 to differentiate into mature tTreg cells. However, one recent study indicated that CD25^+^ Foxp3^-^ tTreg precursors and Foxp3^+^ CD25^-^ tTreg precursors are generated through two distinct developmental programs (30), suggesting that besides TCR/CD28 co-stimulation, some other key factors must be involved during development of these two tTreg precursor populations. All this evidence shows that this model still needs further refinements.

Besides TCR/CD28 co-stimulation, the most probable factors that mediate the distinct developmental programs of tTreg cell are different cytokines. Other than IL-2 and IL-15, three members of the tumor necrosis factor (TNF) superfamily cytokines (GITRL, OX40L and TNF-α) were demonstrated to promote tTreg generation (31). Moreover, TGF-β has also been shown to be important for tTreg cell generation (25, 32). In this review, we summarize the existing studies showing the important functions of cytokines in tTreg cell generation. We conclude that IL-2, IL-7, IL-15, IL-4, TGF-β, GITRL, OX40L, and TNF-α all play important roles in regulating tTreg cell generation, although regulation mechanisms of these cytokines have yet to be confirmed.

## Four γc Family Cytokines (IL-2, IL-7, IL-15 and IL-4)

### Function of IL-2, IL-7, IL-15 and IL-4 in tTreg Cell Generation

Before Treg cells were well identified, it was determined that mice deficient in IL-2 (33–35), IL-2 receptor α chain (IL-2Rα, also called CD25) (36) or IL-2 receptor β chain (IL-2Rβ, also called CD122) (37) would develop severe autoimmunity. It was a surprising finding since IL-2 was found to be a critical T cell growth factor (38–40). Since Treg cells have been identified, CD25 was proven to be a surface marker of Treg cells (2), and then it was determined that Treg cell-deficient scurfy mice develop severe autoimmunity as well (3–5, 41). These findings suggested that IL-2 might play a vital role in Treg cell generation.

However, the function of IL-2 in tTreg cell generation is still contentious. Some studies are against the idea that IL-2 is key for tTreg cell generation, because a significant number of CD4^+^ CD8^-^ CD25^-^ FOXP3^+^ thymocytes were still present in IL-2 knockout (*Il2* ^-/-^) mice, and these cells could still suppress inflammation in adaptive transfer mice model (42–44), although CD25^-^ FOXP3^+^ thymocytes were defined as tTreg precursors in the two-step model (29). Moreover, a recent study found that IL-2 could modulate the tTreg cell epigenetic landscape by targeting genome wide chromatin accessibility (45). These studies showed that IL-2 is dispensable for tTreg cell development, but important for mature tTreg cell survival, tTreg cell stabilization, and tTreg cell suppression function. Consistent with this idea, it was determined that Foxp3 is a proapoptotic protein and these Foxp3^+^ CD25^-^ tTreg precursors completed for the limited IL-2 to support their survival (28). In contrast, some studies found that although mice deficient in IL-2 or IL-2Rα had a certain number of Foxp3+ cells, their tTreg cells were not mature, and mice deficient in IL-2Rβ were shown to have a significant decrease in Treg numbers (44, 46), suggesting IL-2 should be important for tTreg cell development. Consistent with this idea, in the two-step model of tTreg cell development, it was found that CD25^+^ FOXP3^-^ tTreg precursors needed IL-2 to convert to mature tTreg cells (26, 27).

**Figure 1:**
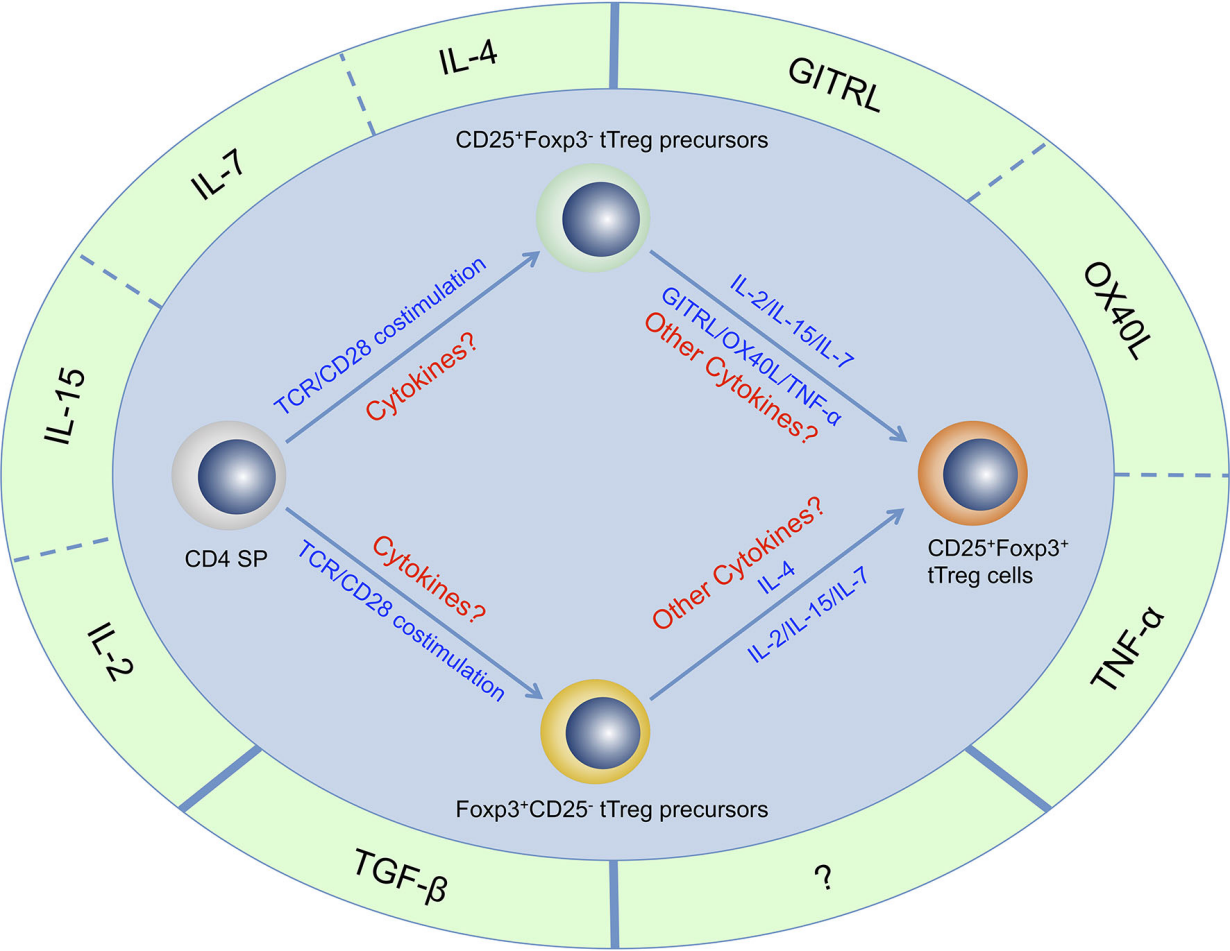
Cytokines that are important for tTreg cell generation. Four γc family cytokines (IL-2, IL-4, IL-7, and IL-15), Three TNF superfamily cytokines (GITRL, OX40L, and TNF-α) and TGF-β have been determined to be important for tTreg cell generation. There may be other cytokines that are important for tTreg cell generation but have not yet been identified. It has been proven that CD4^+^CD8^-^Foxp3^-^CD25^+^ thymocytes and CD4^+^CD8^-^Foxp3^+^CD25^-^ thymocytes are two populations of tTreg cell precursors that generated through two distinct developmental programs, but the regulatory network of these cytokines in the development of these two precursor populations and mature tTreg cell has not been fully revealed.

IL-2 receptor γ chain (IL-2Rγ), also known as the common cytokine receptor γ chain (γc) or CD132, is a common component of the receptors for IL-2, IL-4, IL-7, IL-9, IL-15, and IL-21 (γc family cytokines) (47, 48). Therefore, besides IL-2, functions of other γc family cytokines in tTreg cell generation have also attracted a lot of attention. Importantly, mice deficient in IL-2Rβ resulted in a large reduction in the number of tTreg cells, whereas mice deficient in IL-2 or IL-2Rα still have high Foxp3 expression (42, 44, 46). IL-2Rβ is the receptor for both IL-2 and IL-15, so the function of IL-15 in tTreg cell generation was determined. Indeed, IL-2 and IL-15 double knockout (*Il2* ^-/-^x*Il15* ^-/-^) mice have a significant decrease in Treg numbers compared with *Il2* ^-/-^ mice (44), showing that IL-2 and IL-15 are important for tTreg cell generation. Moreover, mice deficient in IL-2Rγ were shown to be devoid of tTreg cells and have no expression of Foxp3 (42, 49), suggesting other γc family cytokines might also be important for tTreg cell generation. After in-depth research and verification, IL-7 was proven to be important for tTreg cell generation (50, 51). Moreover, IL-2Rβ and IL-7 receptor subunit α (IL-7Rα, also known as CD127) double knockout (*Il2rb* ^-/-^x*Il7ra* ^-/-^) mice were also devoid of tTreg cells, just like mice deficient in IL-2Rγ (50). Further studies proved that IL-2, IL-7, and IL-15 induces STAT5 phosphorylation and this process is indispensable for tTreg cell generation (49, 50), as STAT5 phosphorylation is critical for tTreg cell development by regulating Foxp3 expression (52–55). Taken together, three γc family cytokines, IL-2, IL-7, and IL-15 are essential for Treg cell generation (**Figure 1**). However, it remains to be confirmed whether these cytokines mainly induce tTreg cell development, promote tTreg cell survival, and/or maintain tTreg cell stabilization.

In the beginning, another γc family cytokine IL-4 was thought to be not important for tTreg cell generation as mice deficient in IL-4 receptor α (IL-4Rα) had absolutely normal tTreg cell generation (50). Moreover, IL-4 was actually shown to suppress Treg cell generation and induce T helper-9 cells (Th9 cells) in periphery and *in vitro* (56–58). However, The same research team corrected the views (59), as they found that IL-4 could promote tTreg cell generation from Foxp3^+^ CD25^-^ tTreg precursors, although IL-4 could not support tTreg cell generation from CD25^+^Foxp3^-^ tTreg precursors (30). This evidence shows that IL-4 plays a role in tTreg cell development from Foxp3^+^CD25^-^ tTreg precursors.

### Source of IL-2, IL-7, IL-15 and IL-4 in the Thymus

Determining the cellular sources of IL-2, IL-7, and IL-15 within the thymus are important in revealing the generation of tTreg cells, and it is also important for autoimmunity treatment through the manipulation of tTreg cells. It has been shown that tTreg cells could not produce IL-2 to support tTreg cell development and survival because Foxp3 represses expression of IL-2 (3, 60). More than that, in IL-2 wild type (*Il2* ^+/+^) and *Il2* ^-/-^ bone marrow chimera mice, tTreg cell generation was totally rectified in *Il2* ^-/-^ thymocytes and these bone marrow chimera mice did not develop autoimmunity (20). Therefore, tTreg cell generation mainly relied on IL-2 produced by non-Treg cells.

Although dendritic cells (DCs) and B cells were shown to be able to produce IL-2, mice that have selectively deleted IL-2 in DCs and B cells had been shown to have normal tTreg cell development and homeostasis (61, 62), showing DCs and B cells are not the major cellular sources of IL-2 in the thymus. In contrast, tTreg cell development was largely impaired in *Il2* ^f/f^ CD4-Cre mice, suggesting T cells are the key cellular source of IL-2 in the thymus (62). Moreover, a recent study determined that cells that secrete IL-2 are predominantly mature CD4^+^ CD8^-^ (CD4SP) thymocytes in the thymus; it has further been identified that IL-2 is mainly produced by self-reactive CD4SP thymocytes through single-cell RNA sequencing analysis (63). This evidence shows that self-reactive CD4SP thymocytes are the major cellular sources of IL-2 in the thymus.

Unlike IL-2, the major cellular sources of IL-7 and IL-15 are not T cells. It was determined that both cortical thymic epithelial cells (TECs) and medullary TECs express high levels of IL-7, and IL-7 expression in cortical TECs is even higher than in medullary TECs (64). However, medullary TECs that highly expressed MHC class II were the major cellular source of IL-15 (65). Interestingly, it is well documented that tTreg cells are mainly generated in the medulla (66–69), suggesting it might be why IL-7 is not as important as IL-2 and IL-15 during tTreg cell generation in thymus. So far, the major cellular source of IL-4 in the thymus has not been determined (30).

## TGF-β

### Function of TGF-β in tTreg Cell Generation

Although it has been determined that TGF-β is the key inducer of Foxp3 in periphery and *in vitro* (70, 71), the function of TGF-β in tTreg cell generation is still in dispute. During early research, TGF-β was thought to be dispensable for tTreg cell development, because TGF-β1 deficient (8-10 days old) mice (*Tgfb1* ^-/-^) had normal frequency of tTreg cell in thymus (72), and T cell-specific TGF-β receptor II-deficient mice (*Tgfbr2* ^f/f^ x CD4-Cre) did not change the frequency of tTreg cell in thymus (12-14 days old mice) either (73, 74). In contrast, it was shown that TGF-β is critical for tTreg cell stabilization and regulatory function (72–74). Although the same research team repudiated their earlier study and thought TGF-β was not important for tTreg cell function and stabilization (75, 76), a recent study determined that TGF-β is critical for tTreg cell function in specific tissue environments, but not important for tTreg cell stabilization (77).

Surprisingly, TGF-β was identified to be important for tTreg cell development by studying tTreg cell generation in 3-5 days old neonatal mice (32, 78). It was shown that deletion of TGF-β receptor I (*Tgfbr1* ^f/f^ x Lck-Cre) in T cells blocks tTreg cell development largely in 3-5 days old neonatal mice, then tTreg cell frequency was recovered and became even higher in thymus of 3-4 weeks old *Tgfbr1* ^f/f^ x Lck-Cre mice than that in WT mice (32). It was then shown that tTreg cell frequency was increased in thymus due to increased tTreg cell proliferation in *Tgfbr1* ^f/f^ x Lck-Cre mice, as thymocytes lacking TGF-β receptor I produced more IL-2 and tTreg cells lacking TGF-β receptor I proliferated much faster in response to IL-2 (32). More importantly, further deletion of IL-2 in *Tgfbr1* ^f/f^ x Lck-Cre mice (*Tgfbr1* ^f/f^ x Lck-Cre x *Il2* ^-/-^) blocked tTreg cell development and expansion totally, as 3-4 weeks old *Tgfbr1* ^f/f^ x Lck-Cre x *Il2* ^-/-^ mice were devoid of tTreg cells as well (32).

The other group also reported a lack of tTreg cells in the thymus of 3-5 days old neonatal *Tgfbr2* ^f/f^ x CD4-Cre mice, but they proposed that this was due to increased tTreg cell apoptosis caused by the deletion of TGF-β signaling (78). Since TGF-β promotes thymocyte cell survival (79), a Treg cell-specific TGF-β receptor I-deficient mice (*Tgfbr1* ^f/f^ x Foxp3-Cre) was generated to determine whether the main function of TGF-β is to promote tTreg cell survival in the thymus (25). Surprisingly, it was found that tTreg cell frequency and number in *Tgfbr1* ^f/f^ x Foxp3-Cre mice did not decrease at all (25), and the aged *Tgfbr1* ^f/f^ x Foxp3-Cre mice had even more tTreg cells (77), showing the main function of TGF-β in tTreg cell generation is not to support tTreg cell survival. Existing mechanism studies have found that Smad3 could bind at the conserved noncoding sequence 1 (CNS1) of Foxp3 enhancer and induce Foxp3 expression (80, 81), but it was argued that Smad3 binding to the foxp3 enhancer was dispensable for tTreg cell development (82). Taken together, these findings show that TGF-β is critical to tTreg cell development, although the exact mechanisms need to be further identified (**Figure 1**).

### Source of TGF-β in the Thymus

Thymocyte apoptosis has been identified to increase by day 2 after birth (83), TGF-β level was found to increase significantly in the thymus by day 3 after birth (25), and tTreg cells were shown to appear in large numbers in the thymus by day 3 after birth (84). This evidence suggests that tTreg cell generation, thymocyte apoptosis, and TGF-β production are highly correlated. Indeed, one study showed that the intrathymic concentration of TGF-β is highly dependent on thymocyte apoptosis (25). However, the major cellular source of TGF-β in the thymus has not been uncovered. Based on the existing studies, TGF-β is likely to be released from two possible cellular sources. The first possible source is apoptotic T cells that release TGF-β directly (85), and the second possible source is phagocytes that release TGF-β after these cells phagocytize apoptotic cells (86, 87).

It is worth mentioning that TGF-β is secreted into the extracellular matrix in an inactive latent form (latent TGF-β) and needs to be activated to produce bioactive TGF-β (88, 89). By now, it has not been determined how TGF-β is activated in the thymus. One possible mechanism for the activation of TGF-β in the thymus is through apoptotic cell-released ROS, as apoptotic thymocytes could release a high level of ROS (85), and ROS has been shown to induce TGF-β activation and promote Treg cell generation in periphery (90–92).

## Three TNF Superfamily Cytokines (GITRL, OX40L and TNF-α)

### Function of GITRL, OX40L, and TNF-α in tTreg Cell Generation

The tumor necrosis factor (TNF) superfamily is a protein superfamily originally produced as type-II transmembrane proteins, but these proteins can function as cytokines once they are cleaved off the cell membrane by metalloproteinases (93). The receptors of the TNF superfamily are tumor necrosis factor receptor superfamily (TNFRSF) (94). It has been determined that CD25^+^ Foxp3^-^ tTreg precursors and mature tTreg cells express high levels of TNFRSF members called Glucocorticoid-induced tumor necrosis factor receptor-related protein (GITR, also known as CD357 or TNFRSF18), OX40 (also known as CD134 or TNFRSF4) and TNFR2 (also known as CD120b or TNFRSF1B) (26, 31, 95). Moreover, it was found that a TNF superfamily member, TNF-α, a ligand of TNFR2, could promote Treg cell expansion *in vivo* (96–98). These findings suggest that the TNF superfamily might be important for tTreg cell generation.

Three TNF superfamily members, GITRL, OX40L, and TNF-α have been identified to promote tTreg cell generation (31, 99). One study reported that deficiency in TNFR2 reduced tTreg cell generation significantly (99). Another study showed that deficiency in all three of the TNFRSF members GITR, OX40, and TNFR2, or neutralization of TNF superfamily members GITRL, OX40L, and TNF-α together, markedly inhibited the generation of tTreg cells (31) (**Figure 1**). It was shown that GITRL, OX40L, and TNF-α could convert CD25^+^ Foxp3^-^ tTreg precursors into mature Foxp3+ Treg cells at very low dose of IL-2 (31), showing these three TNF superfamily members promote tTreg cell mature from CD25^+^ Foxp3^-^ tTreg precursors. However, it is still not clear whether TNF superfamily members and IL-2 complement each other, or TNF superfamily members just function as compensatory signals of IL-2 signal.

### Source of GITRL, OX40L and TNF-α in the Thymus

Although the major cellular sources of GITRL, OX40L, and TNF-α have not been well defined, it was identified that medullary TECs expressed GITRL, OX40L, and TNF-α, while conventional dendritic cells (cDCs) and plasmacytoid dendritic cells (pDCs) expressed only GITRL and TNF-α (31). Further studies are needed to determine which kind of APCs are the major cellular source of GITRL, OX40L, and TNF-α. Moreover, whether membrane-bound or soluble GITRL, OX40L, and TNF-α play a more important role in tTreg cell generation has not yet been determined either.

## Conclusions and Future Perspective

By summarizing the existing studies of cytokines in tTreg cell generation, we conclude that four members of γc family cytokines (IL-2, IL-4, IL-7 and IL-15), transforming growth factor β (TGF-β), and three members of TNF superfamily cytokines (GITRL, OX40L, and TNF-α) play vitally important roles in regulating tTreg cell generation, although regulation mechanisms of these cytokines have yet to be confirmed. Functions of these cytokines in tTreg cell generation are still divisive. For example, opinions are still divided on the functions of TGF-β and IL-2, whether they are important for tTreg cell development, survival, and/or proliferation are still controversial.

On the other hand, when and how cytokines interact with each other and mediate tTreg cell generation in the thymus remains to be fully revealed. Also, when and how these cytokines take effect during tTreg cell development is still unclear. Therefore, future studies should focus on why developing tTreg cells are divided into two populations of tTreg precursors. Since CD25^+^ Foxp3^-^ tTreg precursors and Foxp3^+^ CD25^-^ tTreg precursors are generated through two distinct developmental programs (30), it is very likely that cytokines play key roles in inducing these two precursor populations besides TCR/CD28 co-stimulation. So far, it has been proven that IL-4 can support tTreg cell generation from Foxp3^+^ CD25^-^ tTreg precursors (30), and TNF superfamily cytokines (GITRL, OX40L and TNF-α) can support tTreg cell generation from CD25^+^ Foxp3^-^ tTreg precursors (31). These findings can partially explain the differences of CD25^+^ Foxp3^-^ tTreg precursors and Foxp3^+^ CD25^-^ tTreg precursors. However, the regulatory network of these cytokines during the development of tTreg precursors and mature tTreg cell has not yet been fully revealed. It is beyond all doubt that answering these basic questions is extremely important for fully disclosing the generation of tTreg cells.

## Author Contributions

MT drafted the manuscript. FJ, FN, FZ, and ZY reviewed and edited the manuscript. DZ supervised the work and wrote the manuscript. All authors contributed to the article and approved it for publication.

## Funding

This work was supported by the National Natural Science Foundation of China (NO. 82171829, 81600876), the Key Project of the Science and Technology Department of Sichuan Province (NO. 2022YFH0100, 2020YFS0210), the 1·3·5 Project for Disciplines of Excellence, West China Hospital, Sichuan University (NO. ZYYC21012), and the Fundamental Research Funds for the Central Universities (NO. 20822041E4084).

## Conflict of Interest

The authors declare that the research was conducted in the absence of any commercial or financial relationships that could be construed as a potential conflict of interest.

## Publisher’s Note

All claims expressed in this article are solely those of the authors and do not necessarily represent those of their affiliated organizations, or those of the publisher, the editors and the reviewers. Any product that may be evaluated in this article, or claim that may be made by its manufacturer, is not guaranteed or endorsed by the publisher.
